# Supplementary material for: Unusual hydrogen implanted gold with lattice contraction at increased hydrogen content
Source: Nat Commun. 2021 Mar 10;12:1560. doi: 10.1038/s41467-021-21842-9 (PMC7946955; doi:10.1038/s41467-021-21842-9)
Supplement: Supplementary file 1 — Supplementary Information [file 41467_2021_21842_MOESM1_ESM.pdf]

Supplementary Information

Unusual hydrogen implanted gold with lattice contraction at increased hydrogen content

Nguyen Khac Thuan[1], Vuong Van Hiep[2], Nguyen The Nghia[2], Nguyen Trong Tinh[3], Tomoyuki Yamamoto[4], Hoang Nam Nhat[1,5,*]

[1] Faculty of Engineering and Nanotechnology, VNU-University of Engineering and Technology, 144 Xuan Thuy, Cau Giay, Ha Noi 10000, Viet Nam

[2] Faculty of Physics, VNU-Hanoi University of Science, 334 Nguyen Trai, Thanh Xuan, Ha Noi 10000, Viet Nam

[3] Institute of Applied Physics and Scientific Instrument, Vietnam Academy of Science and Technology, 18 Hoang Quoc Viet, Ha Noi 10000, Viet Nam

[4] School of Fundamental Science and Engineering, Waseda University, Shinjuku, Tokyo 169-8555, Japan

[5] Advanced Institute of Engineering and Technology, VNU-University of Engineering and Technology, 144 Xuan Thuy, Cau Giay, Ha Noi 10000, Viet Nam

[*]Corresponding author's email: namnhat@gmail.com

1. Images of sample surface

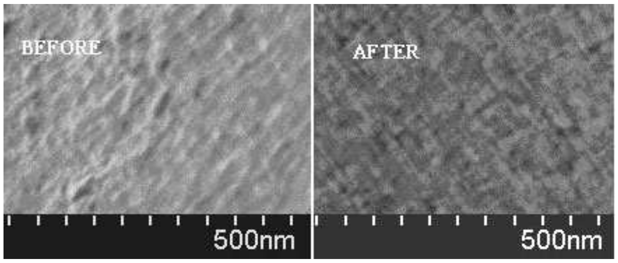

Supplementary Figure 1. Surface of a 560 appm (100 μC) hydrogen implanted sample before and after implantation. The visual difference (the change of colour) between the undoped and the implanted samples is obvious.

2. Model structures of supercells

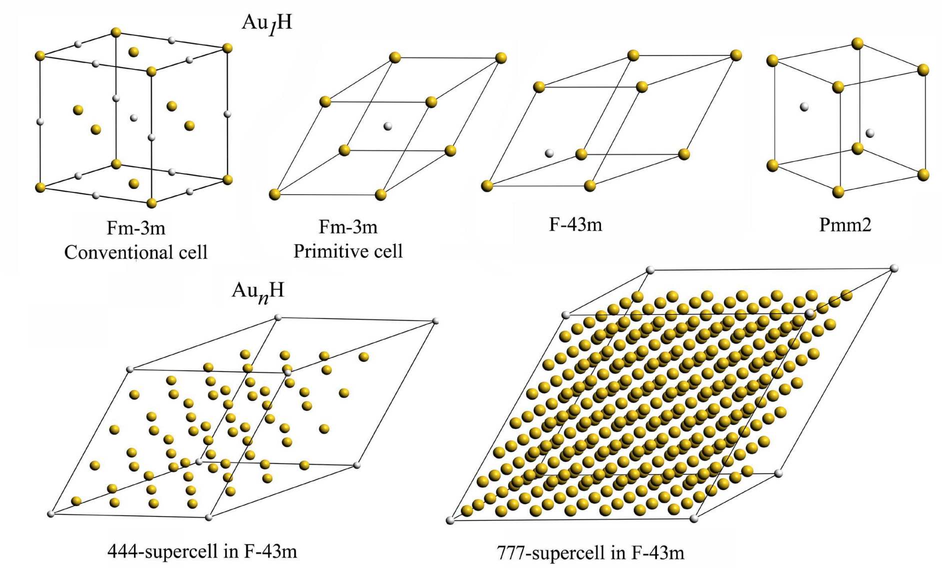

Supplementary Figure 2. Selected models of H-doped supercells.

The structure of gold hydride AuH (may be considered as 100% H-doped, Au_1H in our notation) in *Fm-3m*: the hydrogen atom occurs at the *bcc* position (1/2, 1/2, 1/2). For faster computation the conventional *Fm-3m* cell is converted into smaller primitive cells with Au and H remaining at the same positions. With this primitive cell, the different displacements of hydrogen from *bcc* position lead to different primitive cells of reduced symmetries (Supplementary Table 1, below): *F–43m* (H at 1/4,1/4,1/4), *Immm* (1/2,1/2,0), etc. The initial *P*1 cell can be created by putting the hydrogen atom at a position close to a tetrahedral one, e.g. at (0.21, 0.18, 0.33). To create a *P*1 supercell of size *nnn* first create a *nnn-F-43m* supercell with hydrogen at (1/4,1/4,1/4) then alter the hydrogen position accordingly. Both LDA[1] and GGA/PBE[2] functionals are used for structure optimization, but only GGA/PBE is used for phonon calculations.

Supplementary Table 1. The positions of hydrogen atom in different space groups and group theory's analysis of phonon modes

S.G. (#)	H-position*)	Wyckoff's symbol, site sym.	H-related phonon modes	Au-related phonon modes**)	Calc. Raman activity (cm^{-1})	Illustration of 111-supercell
Fm-3m (225)	1/2, 1/2, 1/2	4b (m-3m)	T_{1u} (Γ_4^+)	A_{1g} (Γ_1^+), A_{1u} (Γ_1^-), A_{2g} (Γ_2^+), A_{2u} (Γ_2^-), E_g (Γ_3^+), E_u (Γ_3^-), T_{2u} (Γ_5^-), T_{2g} (Γ_5^+), T_{1u} (Γ_4^-), T_{1g} (Γ_4^+)	not active	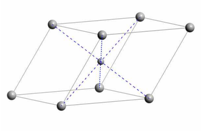
F-43m (216)	1/4, 1/4, 1/4	4d (-43m)	T_2 (Γ_5)	A_1 (Γ_1), A_2 (Γ_2), E (Γ_3), T_1 (Γ_4), T_2 (Γ_5)	352	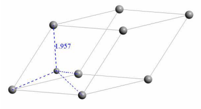
R3m (160)	x(1/4,1/4, 1/4) $x \approx 1$	3a (3m)	A_1 (Γ_1), E (Γ_3)	A_1 (Γ_1), A_2 (Γ_2), E (Γ_3)	241, 835, 838	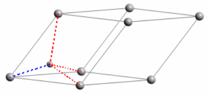
Immm (71)	1/2, 1/2, 0	2b (mmm)	A_g (Γ_1^+)	A_g (Γ_1^+), B_{1g} (Γ_3^+), B_{2g} (Γ_2^+), B_{3g} (Γ_4^+), A_u (Γ_1^-), B_{1u} (Γ_3^-), B_{2u} (Γ_2^-), B_{3u} (Γ_4^-)	1381	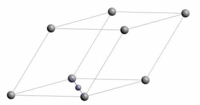
Cmmm (65)	0,0,1/2	2d (mmm)	A_g (Γ_1^+)	A_g (Γ_1^+), B_{1g} (Γ_3^+), B_{2g} (Γ_2^+), B_{3g} (Γ_4^+), A_u (Γ_1^-), B_{1u} (Γ_3^-), B_{2u} (Γ_2^-), B_{3u} (Γ_4^-)		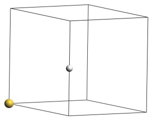
Imm2 (44)	x/2,1/2,1/2 $x \approx 1$	2b (mmm)	A_1 (Γ_1), B_1 (Γ_2), B_2 (Γ_4)	A_1 (Γ_1), A_2 (Γ_3), B_1 (Γ_2), B_2 (Γ_4)	352 --- ---	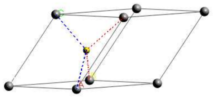
Pmm2 (25)	1/2,0, z/2 $z \approx 1$	1c (mm2)	A_1 (Γ_1), B_1 (Γ_2), B_2 (Γ_4)	A_1 (Γ_1), A_2 (Γ_3), B_1 (Γ_2), B_2 (Γ_4)	444 799 935	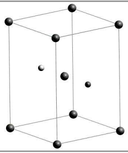
P1 (1)	x/4, y/4, z/4 ($x \neq y \neq z$)$\approx$1	1a (1)	3A (Γ_1)	A (Γ_1)	315, 778, 1113	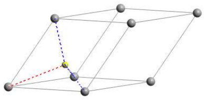

*) The coordinates are given for *111*-supercell and should be divided by the size n of the supercell for the larger, *e.g.* in the *222*-supercell there should be H-coordinates (1/4, 1/4, 1/4) in *Fm-3m*, (x/4, 1/4, 1/4) in *Imm2*, etc.

**) The number of Au-related phonon modes increases with the size n of supercells. In general there are $3n^3-3$ Au-related optical phonon bands in the *nnn*-supercell.

3. Map of electron densities for the *222*-supercell

It may be useful to show the interatomic force constant (IFC) for some model structures as it directly relates to bonding of hydrogen in the Au host lattice, but as far as IFC decays very fast it will be better to show the map of electron density by isosurfaces to reveal the places in the lattice of equal densities. In Supplementary Figure 3, the red color corresponds to higher values of density and the blue to the smaller ones. The density field is set at 80% transparency to show the densities of the inner layers. It appears clearly that hydrogen is bound only to the nearest neighbour Au atoms with electron density of the bonds close to that of the surrounding free spaces, that is, of the conduction band. This illustrates the delocalized nature of the hydride bonds formed between hydrogen and the gold lattice.

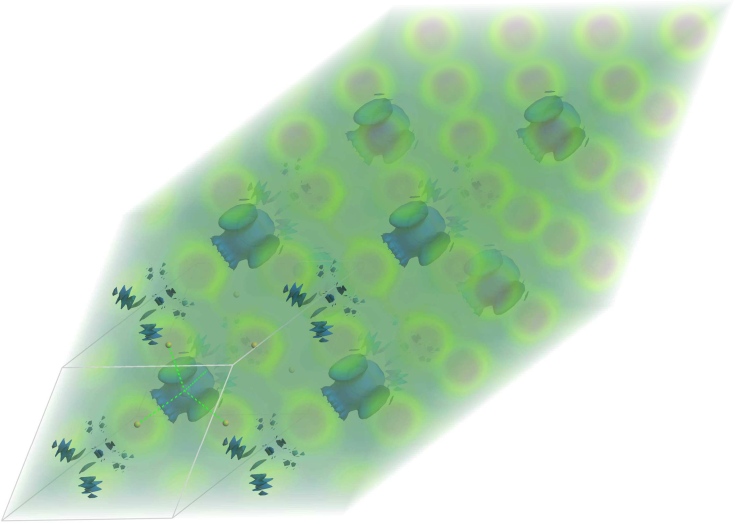

Supplementary Figure 3. Map of electron density for the *222*-supercell.

4. Remarks on phonon calculation and anharmonicity

As a common feature of all DFT codes, CASTEP calculates an interatomic force constant (IFC) matrix individually for each pair of displacement and polarization vectors throughout the Brillouin zone. From IFC it constructs a dynamical matrix by Fourier transformation and from the dynamical equation it determines the phonon frequencies ω and proper eigenvectors of displacements. The mode Grüneisen parameters γ_i can be obtained for all phonon modes by simulating phonons in successive relaxed structures with small differences in volume. In cases of negative lattice expansion, the contribution of the negative γ_i is usually large. Using the *nnn*-supercells with only one hydrogen atom inserted, the phonon dispersion curves were divided into two different groups, of which the 3 upper bands (LO, TO_1 and TO_2) belong to the motions of hydrogen, while the remaining $3n^3$ bands belong to Au. Since there are only 3 acoustic bands, the rest $3n^3\text{−}3$ bands correspond to the Au optical resonances, *i.e.,* Au atoms move in the opposite directions. It is useful to reveal that all phonon bands associated with Au are below 200 cm^{-1}. Remarkably, since the interatomic force constants decay rapidly as R^{-5} and become negligible at Au-H distances

greater than 2.7 Å, it is not necessarily necessary to consider very large supercells; indeed, the *111*- and *222*-supercells are sufficient to simulate the correct dispersion curves. The obtained results show that at higher symmetries ($m\bar{3}m$, $\bar{4}3m$), the mode Grüneisen parameters are all positive, but when the symmetry decreases to *Cmmm, Pmm2* and *P*1 the TO_1, TO_2 phonon bands begin to divide. The large negative mode Grüneisen parameters appear in the directions of polarization vectors that are parallel to the axis connecting the two Au atoms creating bonds with the hydrogen atom. The anharmonicity arises as a result of the asymmetric splitting of modes due to the shortening of one Au-Au axis. The splitting of phonons implies for the observed Raman intensities that, the two peaks at 1091 and 1125 cm^{-1} are the *P*1 splits of a mode at 1381 (A_g, *Immm*) and 815 and 910 cm^{-1} are the *Pmm*2 splits of a mode at 837 cm^{-1} (A_1, *R*3m). The mode at 631 cm^{-1} is a two-phonon state of a *P*1 version (315 cm^{-1}) of an A_{1g} mode (352 cm^{-1}) in $F\bar{4}3m$. Experimentally, the modes with larger mode Grüneisen parameters are 460 and 1125 (positive) and 631 and 1091 cm^{-1} (negative).

Supplementary References

[1] Perdew, J. P., Zunger, A. Self-interaction correction to density-functional approximations for many-electron systems. *Phys. Rev. B* 23, 5048-5079 (1981).

[2] Perdew, J.P., Burke, K., Ernzerhof, M. Generalized Gradient Approximation Made Simple. *Phys. Rev. Lett*. 77, 3865-3868 (1996).
