# Supplementary material for: Unusual hydrogen implanted gold with lattice contraction at increased hydrogen content
Source: Nat Commun. 2021 Mar 10;12:1560. doi: 10.1038/s41467-021-21842-9 (PMC7946955; doi:10.1038/s41467-021-21842-9)
Supplement: Supplementary file 8 — Description of Additional Supplementary Files [file 41467_2021_21842_MOESM8_ESM.pdf]

Description of Additional Supplementary Files

**File**: Supplementary Data 1

**Description**: Data of x-ray and Hall measurements

**File**: Supplementary Data 2

**Description**: Data of Raman and UV-Vis measurements

**File**: Supplementary Data 3

**Description**: Calculated structures by Density Functional Theory

**File**: Supplementary Data 4

**Description**: Band structure, density of states and absorption spectra

**File**: Supplementary Data 5

**Description**: Calculated phonons and mode Gruneisen parameters
